# Manipulation of an α-glucosidase in the industrial glucoamylase-producing *Aspergillus niger* strain O1 to decrease non-fermentable sugars production and increase glucoamylase activity

**DOI:** 10.3389/fmicb.2022.1029361

**Published:** 2022-10-20

**Authors:** Wenzhu Guo, Dandan Liu, Jingen Li, Wenliang Sun, Tao Sun, Xingji Wang, Kefen Wang, Qian Liu, Chaoguang Tian

**Affiliations:** ^1^Key Laboratory of Systems Microbial Biotechnology, Tianjin Institute of Industrial Biotechnology, Chinese Academy of Sciences, Tianjin, China; ^2^National Technology Innovation Center of Synthetic Biology, Tianjin, China; ^3^State Key Laboratory of Agrobiotechnology and MOA Key Laboratory of Soil Microbiology, College of Biological Sciences, China Agricultural University, Beijing, China; ^4^Longda Biotechnology Inc., Shandong, China

**Keywords:** α-glucosidase, transglycosylation activity, *Aspergillus niger*, glucoamylase, reducing sugars

## Abstract

Dextrose equivalent of glucose from starch hydrolysis is a critical index for starch-hydrolysis industry. Improving glucose yield and decreasing the non]-fermentable sugars which caused by transglycosylation activity of the enzymes during the starch saccharification is an important direction. In this study, we identified two key α-glucosidases responsible for producing non-fermentable sugars in an industrial glucoamylase-producing strain *Aspergillus niger* O1. The results showed the transglycosylation product panose was decreased by more than 88.0% in *agdA*/*agdB* double knock-out strains than strain O1. Additionally, the B-P1 domain of agdB was found accountable as starch hydrolysis activity only, and B-P1 overexpression in Δ*A*Δ*B*-21 significantly increased glucoamylase activity whereas keeping the glucoamylase cocktail low transglycosylation activity. The total amounts of the transglycosylation products isomaltose and panose were significantly decreased in final strain B-P1-3 by 40.7% and 44.5%, respectively. The application of engineered strains will decrease the cost and add the value of product for starch biorefinery.

## Introduction

Starch is one of the most important polysaccharides and is abundant in plant tuber and grains (Vanier et al., 2017). As an important industrial feedstock, starch and its hydrolysates have many applications in food and non-food industries (Adewale et al., 2022), including in the production of glucose and/or fructose syrups (Sun et al., 2014), as thickening agents and fat substitutes (Payne et al., 2011), and in the paper, textile, pharmaceutical, cosmetics, and packaging industries (Hashim, 2020; Maniglia et al., 2021). Acid hydrolysis of starch has been substituted by enzymatic hydrolysis because of its environmental and economic benefits (de Souza et al., 2019). The enzymes used in the starch hydrolysis industry are mainly glucoamylase, α-amylase, α-glucosidase, and β-amylase (Vihinen and Mantsala, 1989; Xu et al., 2018). These enzymes are predominantly produced by microorganisms, such as *Bacillus subtilis*, *Aspergillus niger* and *A. oryzae* (Kim et al., 2008; Guo et al., 2021; Ichikawa et al., 2021). Many organisms have been engineered to improve the production of amylolytic enzymes for starch hydrolysis (Wong et al., 2007; van Zyl et al., 2012; Parashar and Satyanarayana, 2017; An et al., 2019). The α-amylase of *Bacillus acidicola* and glucoamylase gene of *Aspergillus niger* were engineered to generate a chimeric biocatalyst in *E*. *coli*, and the biocatalyst saccharified wheat and corn starches more efficiently (Parashar and Satyanarayana, 2017). Barley α-amylase and *lentinula edodes* glucoamylase expressed in *saccharomyces cerevisiae* synergistically enhanced the rate of hydrolysis by three times for corn and wheat starch granules, compared to the sum of the individual activities (Wong et al., 2007). The glucoamylase activity increased significantly from 28250 U/ml to 40710 U/ml after overexpression of *amyA* and *glaA* in *Aspergillus niger* with 1% casein phosphopeptides added to the fermentation medium (An et al., 2019).

With a mechanism of action similar to that of glucoamylase, α-glucosidase is an exoenzyme catalyzing the hydrolysis of α-glycosidic linkages at the non-reducing terminal of substrates *via* an anomer-retaining reaction mechanism (Saburi et al., 2015). This enzyme is involved in the utilization of starch and oligosaccharides by many organisms (Ma et al., 2019) and can be of animal, plant, bacterial, and fungal origin (Tomasik and Horton, 2012). According to the CAZy database, α-1, 4-glucosidases can be grouped into several glycoside hydrolase (GH) families: 4, 13, 31, 63, 97 and 122 (Fujimoto et al., 2017). α-Glucosidases are mainly classified into the glycoside hydrolase families 13 and 31 (Ma et al., 2019).

α-Glucosidases are widely distributed in animals, plants and microbes (Chiba and Shimomura, 1975; de Burlet et al., 1979; Nigam and Singh, 1995). Because fungi depend on their ambient environment for nutrition, they produce α-glucosidases as endocellular and extracellular enzymes (Ichikawa et al., 2021). The intracellular α-glucosidase MalT in *A. oryzae* can convert maltose to isomaltose by transglycosylation, thereby controlling the activation of the transcription factor amyR that is essential for amylolytic gene expression (Ichikawa et al., 2021). α-Glucosidases play important roles in fermentation and have been used to achieve desirable tastes in miso by producing large amounts of isomaltooligosaccharides (Kawano et al., 2021). The transglycosylation activity of α-glucosidases allows them to produce a variety of oligosaccharides, such as isomaltooligosaccharides (α-1, 6-glucooligosaccharides), nigerooligosaccharides (α-1, 3-glucooligosaccharides), and kojibiose (α-1, 2-glucobiose) (Ma et al., 2019). These oligosaccharides have many benefits such as prebiotic functions, preventing tooth decay, ameliorating hepatic dysfunction caused by arachidic acid and increasing iron absorption (Ma et al., 2019).

Although these oligosaccharides have many benefits for humans, the generation of transglycosylated products is undesirable in the glucose industry and during citric acid fermentation (Wang et al., 2016). For cost reasons, corn starch is used instead of glucose in the industrial production of enzymes and organic acids by microorganisms (Dao et al., 2013; Meng et al., 2022). The filamentous fungus *A. niger* secretes a variety of hydrolytic enzymes such as α-amylase, glucoamylase, and α-glucosidase that hydrolyzes starch to generate glucose (Guo et al., 2021). The transglycosylation activity of α-glucosidase results in the accumulation of non-fermentable oligosaccharides such as isomaltose, isomaltotriose, and panose during glucose production. The presence of transglycosylated products reduces the quality of glucose and affects its crystallization (Li et al., 2014). The presence of non-fermentable transglycosylated products also reduces the production of organic acids, including citric acid (Wang et al., 2016).

To improve substrate utilization and decrease non-fermentable sugars production, the genetic basis of transglycosylation should be explored in glucoamylase-producing industrial strains. In this study, two α-glucosidase genes *agdA* and *agdB* were identified responsible for transglycosylation in the industrial glucoamylase-producing *A. niger* strain O1. The transglycosylation activity decreased significantly in the *agdA*/*agdB* double KO strains than in O1. Additionally, the specific domain B-P1 was identified responsible for starch hydrolysis activity in *agdB*. Overexpression of B-P1 domain in strain Δ*A*Δ*B*-21 significantly increased glucoamylase activity. The transglycosylation products isomaltose and panose decreased significantly in B-P1 strain compared with strain O1 in products detection of starch liquefaction and saccharification. The application of the engineered strains will decrease the cost and add the value of product for starch biorefinery.

## Materials and methods

### Strains, medium and growth conditions

The *Aspergillus niger* strain O1, an industrial glucoamylase-producing strain with an aconidial phenotype, was provided by Longda Biotechnology Inc. (Shandong, China). Strain O1 and the transformants were cultured at 34°C on Czapek-Dox solid medium. The composition of Czapek-Dox solid medium (per 100 ml) was as follows: sucrose 3 g, NaNO_3_ 0.2 g, MgSO_4_⋅7H_2_O 0.102 g, KCl 0.05 g, FeSO_4_⋅7H_2_O 0.00183 g, K_2_HPO_4_ 0.1 g, agar 1.5 g, pH 6.0. The semisolid medium, which was used to prepare mycelia for fermentation, had the same composition with agar concentration 0.04% (w/v). The components of the shake-flask fermentation medium were 100 g/L glucose, 30 g/L soybean flour and 30 ml/L corn steep liquor, pH 5.6. Equal weights of mycelia were each inoculated into 50 ml fermentation medium in 250-ml Erlenmeyer flasks. Then, the shake flasks were cultured at 34°C with shaking at 240 rpm for 6 days. Samples of the fermentation broth were collected at 2, 4, and 6 days of cultures for extracellular protein, secretome, and glucoamylase activity analyses. For plasmid proliferation, *Escherichia coli Tran1*-T1 was cultured at 37°C in Luria-Bertani (LB) medium supplemented with kanamycin (50 mg/L) or ampicillin (100 mg/L). The components of LB medium were as follows: tryptone 10 g/L, yeast extract 5 g/L, and NaCl 10 g/L.

### Construction of plasmids for deletion of α-glucosidases and overexpression of specific domains

The primers used in this study are listed (Supplementary Table 1). For the deletion of *agdA* (An04g06920) and *agdB* (An01g10930), gene-deletion cassettes were constructed (Supplementary Figure 2A). The 5′ and 3′ flanking sequences of An04g06920 and An01g10930 were amplified using the primers A-donor-F1/R1 and A-donor-F3/R3, and B-donor-F1/R1 and B-donor-F3/R3, respectively. PtrpC-neo cassettes were amplified from P0380-neo using the primers A-donor-F2/R2 and B-donor-F2/R2 for deletion of *agdA* and *agdB*, respectively. The fragment 5′-PtrpC-neo-3′ was fused by overlapping PCR and cloned into the Pjet1.2/blunt cloning vector to generate donor DNA sequences. Specific sgRNAs targeting *agdA* and *agdB* were designed using the sgRNACas9 tool and sgRNA target sites with high scores were selected. All protospacer sequences used to target the genes are listed in Supplementary materials. The U6 promoter was amplified from the genome of *A. niger* O1. sgRNAs and sgRNA scaffold fragments were fused by overlapping PCR. The final resulting fusion fragments were cloned into the Pjet1.2/blunt cloning vector for sequencing. The Cas9-expression PCR cassette AnPtef-Cas9-TtrpC was amplified from the plasmid P0380-AnPtef-Cas9-TtrpC, which was constructed in our laboratory, using the primers An-Ptef/cas9-F/R.

According to the protein domain information of agdB, the DNA sequences of B-P1, and B-P2 were amplified from the genome of *A. niger* O1 using the primers B-3amp-F/B-P1-R and B-3amp-F/B-P2-R, respectively. The DNA sequence of B-P3 was amplified using the primers B-3amp-F/B-P3-link-R and B-P3-link-F/B-P3-R, and the two parts of B-P3 were fused by overlapping PCR. The sequences of B-P1, B-P2 and B-P3 were each cloned into the Pjet1.2/blunt cloning vector for sequencing. Then, the identified fragments were integrated to the *Eco*RV-digested PAN52-AnPgpdA-TtrpC vector using the Gibson Assembly^®^ Cloning Kit (NEB, Beverly, MA, United States) to complete the construction of the OE plasmids.

### Protoplast transformation of *Aspergillus niger*

Protoplast transformation of *A. niger* was carried out as described previously with some modifications (Wang et al., 2015). To overexpress specific protein domains, 10 μg linearized plasmid was mixed with the *A. niger* protoplasts. For gene disruption by the CRISPR/Cas9 system, 10 μg Cas9-expression cassette AnPtef-Cas9-TtrpC, sgRNA expression cassette, and the corresponding donor fragments were mixed at a molar concentration ratio of 1:1:1 and then added to *A. niger* protoplasts (Liu et al., 2017). The transformants were cultured on Vogel’s MM solid medium at 34°C for 4 days with selection for resistance to geneticin (250 mg/L) or hygromycin (100 mg/L). The strains overexpressing specific protein domains or with disrupted *agdA*/*agdB* were identified by diagnostic PCR.

### Total RNAs extraction and quantitative real-time PCR

The mycelia were harvested from 2-day, 4-day, and 6-day shake-flask cultures by vacuum filtration, and then homogenized in liquid nitrogen for total RNA extraction. Total RNA was isolated and purified using a Qiagen RNeasy Mini Kit (Qiagen, Hilden, Germany). Total RNA was synthesized to first strand cDNA using ReverTra Ace qPCR RT Kit (TOYOBO, Osaka, Japan). qRT-PCR was performed using SYBR Green Real-time PCR Master Mix (TOYOBO, Osaka, Japan). The primers used for detection of expression of α-glucosidases are listed (Supplementary Table 1). The *actin* gene An15g00560 was used as an internal control. The expression level of each gene was estimated using the 2^–ΔΔ*CT*^ method (Livak and Schmittgen, 2001).

### Glucoamylase activity assay

Because extracellular protein level and total glucoamylase activity are highest in 6-day supernatant, the enzyme activity and secretome of 6-day supernatants were determined and analyzed. Glucoamylase activity was determined by a modified 3,5-dinitrosalicylic acid method using soluble starch as the substrate. Firstly, the fermentation supernatants were appropriately diluted using 50 mM sodium acetate buffer, pH 4.6. Then, 250 μL 1% (w/v) soluble starch preheated in a water bath at 62°C for 5 min was added to 250 μL diluted supernatant and mixed well. The mixture was incubated at 62°C for a further 10 min and then the reaction was terminated by adding 500 μL 3,5-dinitrosalicylic acid. The samples were boiled for 10 min and cooled on ice for 5 min before adding 1.5 ml distilled water. The glucoamylase activity was evaluated by calculating the amount of reducing sugars released from starch hydrolysis from the absorbance at 540 nm.

### Transglycosylation activity assay

To decrease glucose production when maltose was used as the substrate in the transglycosylation activity assay, 0.75 g/L acarbose was used to inhibit the activity of amylolytic enzymes as described in previous work (Li et al., 2014). Firstly, 0.75 g/L acarbose was prepared using 20 mM sodium acetate buffer pH 4.8. Then, 0.4 ml 0.75 g/L acarbose was mixed with 0.1 ml 20 times diluted supernatant, which was preheated at 37°C for 10 min. Next, 0.5 ml 20% maltose prepared using 20 mM sodium acetate buffer, pH 4.8, was added to the reaction mixture. The mixture was incubated at 37°C for 24 h, and then the reaction was terminated by boiling for 10 min. The mixture was cooled to room temperature, then mixed with 9 ml 10 mM H_2_SO_4_ before filtering through 0.22 μm PES membrane (Millipore, Billerica, MA, United States). The filtered supernatant was analyzed by high-performance liquid chromatography (HPLC) with an e2695 instrument (Waters, Manchester, United Kingdom) equipped with a Waters 2414 refractive index detector and an Aminex HPX-87H column (Bio-Rad, Hercules, CA, United States). The mobile phase was 10 mM H_2_SO_4_ with a constant flow rate of 0.5 ml/min.

For starch liquefaction and saccharification, liquid α-amylase (kindly provided by Ms. Xiaoling Mu) was used for starch liquefaction and the supernatants produced by O1 and variety of transformants were used for starch saccharification. Before the experiment was conducted, corn starch was dried to constant weight at 60°C. First, 100 μL liquid α-amylase was added to a 30 ml slurry of 30% w/v corn starch (Solarbio, Beijing, China) with 0.2% CaCl_2_ and the mixture was incubated at 85°C for 2 h. The end point of starch liquefaction was identified using 0.05 M I_2_-KI standard solution. The reaction mixture was boiled for 10 min to inactivate the enzyme and then cooled to room temperature. Subsequently, the pH was adjusted to pH 4.4 to provide a favorable environment for glucoamylase activity. A 4.5-ml aliquot of the starch liquefaction product mixture was mixed with 300 μL fermentation supernatant (containing excessive amounts of enzymes) from O1 or each of the transformants. The mixture was then incubated at 62°C for 48 h. Ethyl alcohol was used to verify the end point of starch saccharification (when no precipitate was generated). Finally, the products of starch saccharification were analyzed by HPLC and the reducing sugars content was measured using an SGD-IV automatic reducing sugar detector (BISD, Shandong, China) with Fehling’s solution.

### Secretome analysis by liquid chromatography-mass spectrometry

*Aspergillus niger* O1 and transformants were cultured in fermentation medium and samples were collected at 2, 4, and 6 days of cultures. The samples were centrifuged and the supernatants were filtered through 0.22 μm PES membrane (Millipore). The protein concentration in the culture supernatant was determined using a Bio-Rad protein assay kit according to the manufacturer’s instructions (Bio-Rad, Hercules, CA, United States). Bovine serum albumin (BSA) was used as the standard, and the absorbance was measured at 595 nm. The supernatant of 6-day cultures was assayed by SDS-PAGE. The subsequent LC-MS/MS analyses and identification of proteins in the secretome were performed as described previously (Guo et al., 2021).

## Results and discussion

### Identification of genes related to transglycosylation activity by transcription levels and secretome analyses

According to the results of previous studies, α-glucosidases generate transglycosylated products in bacteria and fungi (Fujimoto et al., 2017; Ma et al., 2017). To identify the genes encoding enzymes catalyzing transglycosylation in *A. niger* O1, an industrial fungal strain, total RNAs were extracted from mycelia collected from 2-day, 4-day, 6-day shake-flask cultures. The transcription levels were detected and analyzed using quantitative real-time PCR. The transcript levels of five predicted α-glucosidase genes were very low, but those of An04g06920 (*agdA*) and An01g10930 (*agdB*) were high in the transcription level analyses of 2-day, 4-day, 6-day cultures (Supplementary Figure 1) (Yuan et al., 2008). On the basis of the results of our previous work, α-glucosidase is the fifth most abundant protein in the secretomes of 2-day, 4-day, 6-day cultures (Guo et al., 2021). Peptide search analyses revealed that A2QAC1 (UniProt accession) was the most abundant α-glucosidase, ranked fifth among all detected proteins in the O1 secretome. A2QAC1 is the translation product of An01g10930, and has α/β-glucosidase agdB annotation in the NCBI database. The gene encoding another predicted α-glucosidase with α-xylosidase activity, A2QTU5, was rarely detected. Surprisingly, although An04g06920 had the highest transcript level of all the α-glucosidase genes, no corresponding protein product was detected in the strain O1 secretome. According to transcription levels and secretome analyses, *agdB* (encoded by An01g10930), the most abundant α-glucosidase in the secretome, and *agdA* (An04g06920), the highest transcript level of all the seven α-glucosidase genes, were selected as the target genes for decreasing transglycosylation activity.

### Knockout of *agdA* and *agdB* decrease transglycosylation activity of α-glucosidases

**FIGURE 1:**
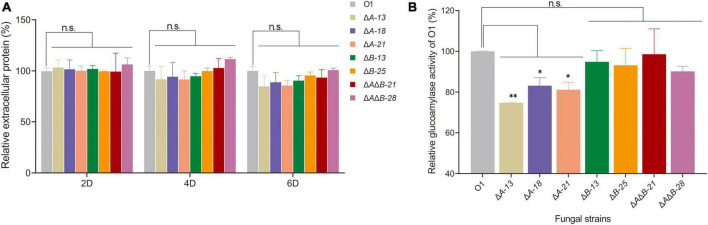
Effects of *agdA* and/or *agdB* knockout (KO) on extracellular protein levels, glucoamylase activity. **(A)** Extracellular protein levels in supernatants of 2-day, 4-day, and 6-day shake-flask fermentation cultures of *Aspergillus niger* O1, *agdA*-KO strains, *agdB*-KO strains, and *agdA*/*agdB* double KO strains. Relative extracellular protein = extracellular protein level in supernatant of engineered strain compared with that in supernatant of strain O1. **(B)** Glucoamylase activity assay in supernatants of 6-day shake-flask fermentation cultures of *A. niger* O1, *agdA*-KO strains, *agdB*-KO strains, and *agdA*/*agdB* double KO strains. Relative glucoamylase activity = glucoamylase activity in supernatant of engineered strain compared with that in supernatant of strain O1. Values are means ± SD (*n* = 3 repeats). ^**^P < 0.01 (Student’s *t*-test), *P < 0.05 (Student’s *t*-test), n.s., not significant.

On the basis of the above analyses, donor DNA and sgRNA cassettes of *agdA* and *agdB* were designed and constructed to knock out *agdA* or *agdB* separately or simultaneously using the CRISPR-Cas9 gene editing system (Supplementary Figure 2A). After protoplasmic transformation, transformants were acquired and identified by diagnostic PCR (Supplementary Figures 2B,C). The extracellular protein levels in the supernatants of 2-day, 4-day, and 6-day fermentation cultures of O1 and transformants were measured. The extracellular protein levels assay showed that the extracellular protein levels of *agdA* KO transformants Δ*A*-13, Δ*A*-18, and Δ*A*-21 were lower than that of strain O1 in the 4-day and 6-day cultures (Figure 1A). The extracellular protein levels in the *agdB* KO transformants Δ*B*-13 and Δ*B*-25 were similar to those in the 2-day, 4-day, and 6-day cultures of strain O1. However, double KO transformants Δ*A*Δ*B*-21 and Δ*A*Δ*B*-28 were slightly higher than that in the 4-day culture and similar to those in 6-day culture of strain O1 (Figure 1A). When the glucoamylase activity of the filtered supernatants was compared between the transformants and O1, the *agdA* KO strains Δ*A*-13, Δ*A*-18, Δ*A*-21 had significantly decreased glucoamylase activity compared with that of O1 (by 25.2%, 16.9% and 18.8%, respectively) (Figure 1B). The glucoamylase activities of the *agdB* KO strains Δ*B*-13, and Δ*B*-25 and double KO strains Δ*A*Δ*B*-21, and Δ*A*Δ*B*-28 were slightly lower than that of O1 (by 5.1%, 6.8%, 2.4% and 9.8%, respectively) (Figure 1B).

The filtered supernatants were also analyzed by LC-MS/MS to detect the protein profiles of 6-day cultures of the KO strains and O1. The secretome of the strains with the *agdB* single KO or *agdA/agdB* double KO were almost the same as that of O1 except for A2QAC1, for which no corresponding peptides were detected (Supplementary material 2).

**FIGURE 2:**
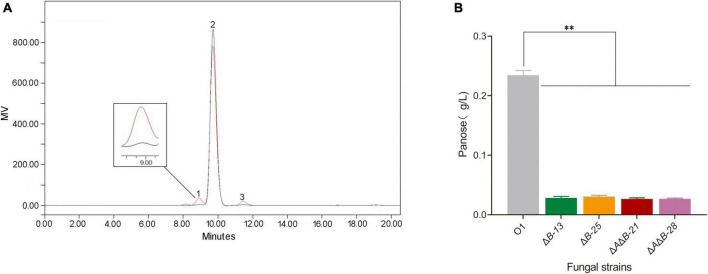
HPLC chromatograms of transglycosylation products and effects of *agdA* and/or *agdB* knockout (KO) on transglycosylation activity. **(A)** HPLC chromatograms of transglycosylation products from 20% maltose as substrate. Peak 1, panose; peak 2, maltose; peak 3, glucose. **(B)** Concentration of sole product, panose, in reaction mixture. Values are means ± SD (*n* = 3 repeats). ^**^P < 0.01 (Student’s *t*-test).

After knocking out single or double genes, the transglycosylation activity of α-glucosidases was detected in a variety of strains. Because the glucoamylase activity was significantly decreased in *agdA* KO strains, subsequent work was conducted in *agdB* KO stains and *agdA*/*agdB* double KO strains. Because *A. niger* strain O1 is an industrial glucoamylase-producing strain, there is a large amount of glucoamylase to hydrolyze α-1, 4-glucosidic linkages, which interferes with the determination of transglycosylation activity (Li et al., 2014). In addition, α-glucosidase is a bifunctional enzyme that also hydrolyzes the α-1, 4-glucosidic linkages of oligosaccharides. Hence, acarbose was used to reduce the interference from hydrolase activity of these amylolytic enzymes. The transglycosylation products of KO strains and O1 were detected by HPLC. The HPLC spectrum revealed three components detected: maltose, panose and glucose (Figure 2A). Of them, panose is the only transglycosylation product. The concentration of panose in reaction mixture of Δ*B*-13, Δ*B*-25, Δ*A*Δ*B*-21, Δ*A*Δ*B*-28 were decreased by 87.9%, 87.0%, 88.6% and 88.5% respectively, compared with that in strain O1 (Figure 2B).

These findings provided further evidence that *agdB* encodes the enzyme responsible for the transglycosylation activity of strain O1, and showed that *agdB* KO and *agdA*/*agdB* double KO significantly decreased transglycosylation activity and slightly decreases glucoamylase activity.

### Identification of the critical domain of *agdB* for starch hydrolysis

**FIGURE 3:**
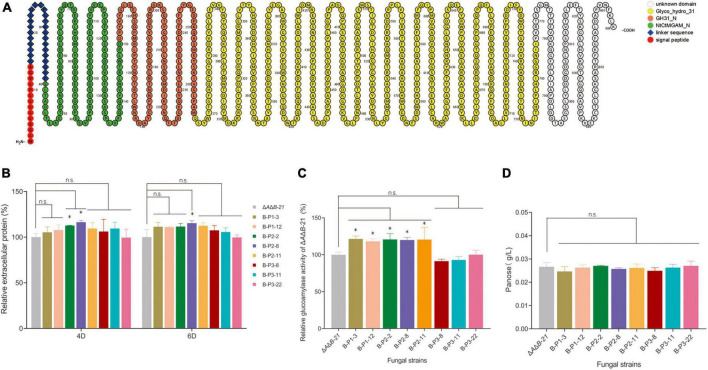
Domain architecture of *A. niger* agdB and effects of overexpressing different domains in strain Δ*A*Δ*B*-21 on extracellular protein levels, glucoamylase activity, and transglycosylation activity. **(A)** Schematic representation of agdB created using Protter protein visualization tool (Omasits et al., 2014). **(B)** Extracellular protein levels in supernatants of 4-day and 6-day shake-flask fermentation cultures of Δ*A*Δ*B*-21 and domain-overexpressing (OE) strains. Relative extracellular protein = extracellular protein level in supernatant of engineered strain compared with that in supernatant of strain Δ*A*Δ*B*-21. **(C)** Glucoamylase activity assay in supernatants of 6-day shake-flask fermentation cultures of Δ*A*Δ*B*-21 and domain-OE strains. Relative glucoamylase activity = glucoamylase activity in supernatant of engineered strain compared with that in supernatant of strain Δ*A*Δ*B*-21. **(D)** Concentrations of sole product, panose, in reaction mixture as detected by HPLC. Values are means ± SD (*n* = 3 repeats). *P < 0.05 (Student’s *t*-test), n.s., not significant.

α-Glucosidase is one of most important enzymes in the last step of starch degradation (Zhou et al., 2009). Blast searches of amino acid sequences revealed three specific protein domains (NtCtMGAM_N, GH31_N, and Glyco_hydro_31) and an unknown functional domain in *agdB* (Figure 3A). A variety of substrates including disaccharides, oligosaccharides, and aryl-and alkyl-α-glucopyranosides can be hydrolyzed by α-glucosidases, which are typically inactive against high molecular weight substrates (Tomasik and Horton, 2012). On the basis of the domain analysis results, we constructed three OE plasmids to identify which domain is responsible for starch hydrolysis activity in *agdB* (Supplementary Figure 3A). The three OE constructs with different domains of *agdB* were designated as B-P1 (containing the signal peptide, linker sequence, and NtCtMGAM_N domain), B-P2 (containing the signal peptide, linker sequence, and NtCtMGAM_N and GH31_N domain), and B-P3 (containing signal peptide, linker sequence, and the Glyco_hydro_31 domain). Δ*A*Δ*B*-21 was selected as the original strain for domain-specific overexpression because it had the highest specific activity of glucoamylase. After protoplast transformation, transformants were acquired and identified by diagnostic PCR (Supplementary Figures 3B–D). The extracellular protein levels in the supernatants of 4-day and, 6-day fermentation cultures of Δ*A*Δ*B*-21 and the transformants were measured. No significant differences in extracellular protein contents were detected between Δ*A*Δ*B*-21 and B-P3 OE transformants in the 4-day and 6-day cultures, but small increases were detected in the supernatants of 4-day and 6-day cultures of B-P1 and B-P2 OE strains (Figure 3B). Subsequently, the glucoamylase activity of supernatants of Δ*A*Δ*B*-21 and domain OE strains 6-day fermentation cultures were tested. The glucoamylase activity was significantly higher in B-P1 and B-P2 OE strains than Δ*A*Δ*B*-21. Compared with the glucoamylase activity in Δ*A*Δ*B*-21, that in B-P1-3 and B-P1-12 was increased by 21.4% and 18.3%, respectively, and that in B-P2-2, B-P2-8, and B-P2-11 was increased by 20.7%, 19.9% and 20.5%, respectively (Figure 3C). However, the glucoamylase activity of the B-P3 OE strains B-P3-8, B-P3-11 and B-P3-22 were almost the same as that of Δ*A*Δ*B*-21 (Figure 3C). The results of transglycosylation activity assays showed that all B-P1, B-P2 and B-P3 OE strains produced panose at comparable levels to that produced by Δ*A*Δ*B*-21 (Figure 3D).

Multiple protein sequence alignment of α-glucosidases confirmed high conserved protein sequences in some regions among *Aspergillus* species (Supplementary Figure 4). Several key elements have been reported to be critical for the transglycosylation activity of α-glucosidases in fungi. An aromatic residue, Tyr296, on β-α loop 1 in the catalytic domain was shown to be important for transglycosylation activity in *A. oryzae* (Supplementary Figure 4; Song et al., 2013; Nagayoshi et al., 2015). This location corresponded to the N-terminal of the Glyco_hydro_31 domain in agdB. The residues 457–560 of *Aspergillus sojae* (*A. sojae*) were found to be the structural basis of transglycosylation activity, corresponding to the middle part of Glyco_hydro_31 domain in agdB (Ding et al., 2022). Mutation of the amino acid residue H450 or R450 in α-glucosidases from *A. oryzae* and *A. sojae* changed the types and amounts of transglycosylation products (Kawano et al., 2021). Similarly, mutation of N696 in *A. niger* α-glucosidase modified transglycosylation properties (Ma et al., 2017).

The above results suggested that NtCtMGAM_N or NtCtMGAM_N and GH31_N may be the domain(s) responsible for starch hydrolysis. Because glucoamylase activity and panose content were unaffected in the B-P3 OE strains, and on the basis of results and information in the literatures, we concluded that the Glyco_hydro_31 domain is an incomplete transglycosylation domain. The whole transglycosylation domain may contain all or part of the GH31_N domain, whole sequence of Glyco_hydro_31, all or some of the domain with unknown function at the C-terminal of agdB.

### Increased reducing sugars production of the B-P1 OE strain by starch degradation under laboratory conditions

**FIGURE 4:**
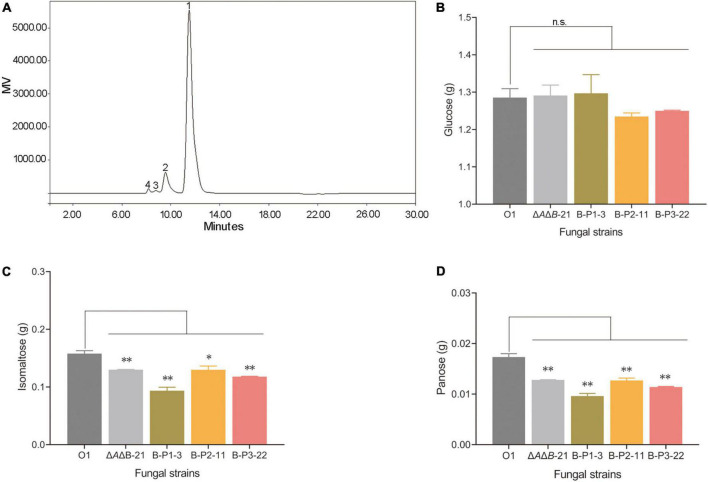
Analysis of starch liquefaction and saccharification products in supernatants of strain O1, Δ*A*Δ*B*-21, and domain-OE strains. **(A)** Analysis of reaction products by HPLC. Peak 1, glucose; peak 2, isomaltose; peak 3, panose; peak 4, unknown product. **(B)** Total amount of glucose detected in reaction mixtures. **(C)** Total amount of isomaltose detected in reaction mixtures. **(D)** Total amount of panose detected in reaction mixtures. Values are means ± SD (*n* = 3 repeats). ^**^P < 0.01 (Student’s *t*-test), *P < 0.05 (Student’s *t*-test), n.s., not significant.

**FIGURE 5:**
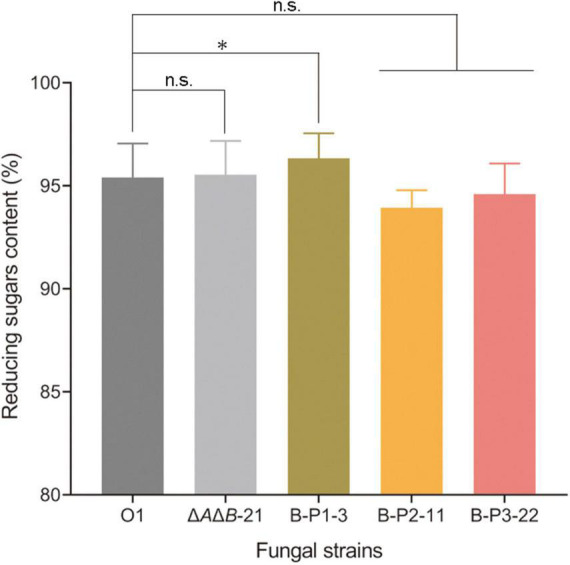
The reducing sugars content of strain O1, Δ*A*Δ*B*-21, and domain-OE strains measured using an SGD-IV automatic reducing sugar detector. Values are means ± SD (*n* = 3 repeats). *P < 0.05 (Student’s *t*-test), n.s., not significant.

*Aspergillus niger* strain O1 is an industrial glucoamylase-producing strain, but the α-glucosidases are also present in fermentation cultures affecting the purity of glucose, and the product of starch degradation. Therefore, two steps of starch liquefaction and saccharification were conducted under laboratory conditions using commercial α-amylase and the supernatants of strains O1, Δ*A*Δ*B*-21, and transformants to assess the effect of genetic manipulation. After starch liquefaction and saccharification, the products were diluted and assayed by HPLC. As shown in Figure 4A, four products were detected: glucose, isomaltose, panose and an unknown component. The unknown component may be a kind of oligosaccharide, but it did not correspond to any of the standards we have and could not be quantified (Supplementary Figure 5). The amounts of the other three products were calculated. Compared with O1, the strains Δ*A*Δ*B*-21 and B-P1-3 produced 0.4% and 0.9% more glucose, respectively (Figure 4B). Compared with O1, the Δ*A*Δ*B*-21 and B-P1-3 strains produced 17.6% and 40.7% less isomaltose, respectively (Figure 4C) and 26.1% and 44.5% less panose, respectively (Figure 4D). Owning to the unknown component of the products, the reducing sugars content was measured using an SGD-IV automatic reducing sugar detector with Fehling’s solution. The reducing sugars content in the product of Δ*A*Δ*B*-21 and B-P1-3 were increased by 0.2% and 0.9% respectively, compared with that in the product of O1 (Figure 5). Thus, *agdB* KO and B-P1 domain overexpression reduced transglycosylation activity and increased the reducing sugars content in the products of starch degradation.

If the GH31_N domain is responsible for starch hydrolysis, then the reducing sugars content of B-P2-11 should have been increased like that of B-P1-3. Instead, it was decreased. These results demonstrated that only the NtCtMGAM_N domain of agdB is responsible for starch hydrolysis. Further research is required to determine which domains are responsible for transglycosylation activity, some or all of the GH31_N domain and Glyco_hydro_31, with or without the domain with unknown function at the C-terminal. Furthermore, the transglycosylation products differed depending on whether maltose or corn starch was used as the substrate. This may be due to the differences in reaction systems. When a high concentration of maltose (20%) was the substrate with the inhibitor acarbose, the product was simple (panose is the sole transglycosylation product). However, when the mixture of starch liquefaction was used as the substrate, its composition was more complex, the concentration of maltose was lower than 20%, and no inhibitor was present in the reaction system. These differences may have led to difference products. In summary, overexpression of the B-P1 domain containing the NtCtMGAM_N domain increased the reducing sugars content, which enhanced the purity of glucose, thereby adding to its value.

## Conclusion

In this study, two α-glucosidase genes *agdA* and *agdB* have been identified responsible for transglycosylation in the industrial glucoamylase-producing *A. niger* strain O1. The transglycosylation product panose was decreased more than 88% in the *agdA*/*agdB* double KO strains than in O1. Additionally, agdB-P1 was identified as the specific domain responsible for starch hydrolysis activity in *agdB*. Overexpression of B-P1 domain of *agdB* in strain Δ*A*Δ*B*-21 significantly increased glucoamylase activity. In products detection of starch liquefaction and saccharification, the transglycosylation products isomaltose and panose decreased significantly as compared with strain O1. The reducing sugars content of the B-P1-3 product increased accordingly. Together, the glucoamylase activity and the glucose content of B-P1 OE strain were increased, as well as the transglycosylated products were decreased. The application of the engineered strains will simplify the production process and decrease the cost of product, increase production of amylose and organic acids, and add the value of product for starch biorefinery.

## Data availability statement

The data presented in this study are deposited in the PRIDE repository (Perez-Riverol et al., 2022), accession number PXD036777 (http://www.ebi.ac.uk/pride/archive/projects/PXD 036777).

## Author contributions

WG contributed to the methodology, data curation, investigation, writing – original draft, and writing – review and editing. DL contributed to the methodology and investigation. JL contributed to the methodology. WS contributed to the project administration. TS contributed to the investigation. XW and KW contributed to the resources and methodology. QL contributed to the methodology, writing – review and editing, supervision, and funding acquisition. CT contributed to the methodology, funding acquisition, writing – review and editing, and supervision. All authors contributed to the article and approved the submitted version.

## References

[B1] Adewale P. Yancheshmeh M. S. Lam E. (2022). Starch modification for non-food, industrial applications: Market intelligence and critical review. *Carbohydr. Polym.* 291:119590. 10.1016/j.carbpol.2022.119590 35698403

[B2] An X. Ding C. Zhang H. Liu T. Li J. (2019). Overexpression of *amyA* and *glaA* substantially increases glucoamylase activity in *Aspergillus niger*. *Acta Biochim. Biophys. Sin.* 51 638–644. 10.1093/abbs/gmz043 31081016

[B3] Chiba S. Shimomura T. (1975). Substrate specificity of flint Corn α-Glucosidase. *Agric. Biol. Chem.* 39 1041–1047.

[B4] Dao T. H. Zhang J. Bao J. (2013). Characterization of inulin hydrolyzing enzyme(s) in commercial glucoamylases and its application in lactic acid production from Jerusalem artichoke tubers (Jat). *Bioresour. Technol.* 148 157–162. 10.1016/j.biortech.2013.08.123 24050923

[B5] de Burlet G. Vannier C. Giudicelli J. Sudaka P. (1979). Neutral α-glucosidase from human kidney. Molecular and immunological properties. Relationship with intestinal glucoamylase. *Biochimie* 61 117–183. 119552

[B6] de Souza I. A. Orsi D. C. Gomes A. J. Lunardi C. N. (2019). Enzymatic hydrolysis of starch into sugars is influenced by microgel assembly. *Biotechnol. Rep.* 22 e00342. 10.1016/j.btre.2019.e00342 31080766 PMC6500924

[B7] Ding Y. Oyagi A. Miyasaka Y. Kozono T. Sasaki N. Kojima Y. (2022). Structural basis for proteolytic processing of *Aspergillus sojae* α-glucosidase L with strong transglucosylation activity. *J. Struct. Biol.* 214:107874. 10.1016/j.jsb.2022.107874 35688347

[B8] Fujimoto Z. Suzuki N. Kishine N. Ichinose H. Momma M. Kimura A. (2017). Carbohydrate-binding architecture of the multi-modular α-1,6-glucosyltransferase from *Paenibacillus* sp. 598K, which produces α-1,6-glucosyl-α-glucosaccharides from starch. *Biochem. J.* 474 2763–2778. 10.1042/BCJ20170152 28698247

[B9] Guo W. Yang J. Huang T. Liu D. Liu Q. Li J. (2021). Synergistic effects of multiple enzymes from industrial *Aspergillus niger* strain O1 on starch saccharification. *Biotechnol. Biofuels* 14:225. 10.1186/s13068-021-02074-x 34838099 PMC8627030

[B10] Hashim S. O. (2020). Starch-modifying enzymes. *Adv. Biochem. Eng. Biotechnol.* 172 221–244. 10.1007/10_2019_91 30937486

[B11] Ichikawa T. Tanaka M. Watanabe T. Zhan S. Watanabe A. Shintani T. (2021). Crucial role of the intracellular α-glucosidase MalT in the activation of the transcription factor AmyR essential for amylolytic gene expression in *Aspergillus oryzae*. *Biosci. Biotechnol. Biochem.* 85 2076–2083. 10.1093/bbb/zbab125 34245563

[B12] Kawano A. Matsumoto Y. Terada A. Tonozuka T. Tada S. Kusumoto K. I. (2021). Modification of the transglucosylation properties of α-glucosidases from *Aspergillus oryzae* and *Aspergillus sojae* via a single critical amino acid replacement. *Biosci. Biotechnol. Biochem.* 85 1706–1710. 10.1093/bbb/zbab091 34014266

[B13] Kim J. C. Kong B. W. Kim M. J. Lee S. H. (2008). Amylolytic hydrolysis of native starch granules affected by granule surface area. *J. Food Sci.* 73 C621–C624. 10.1111/j.1750-3841.2008.00944.x 19021791

[B14] Li S. Feng S. Chen Y. Li T. (2014). Determination of activity of transglycosidase in diastatic enzyme by high performance liquid chromatography. *Se Pu* 32 539–542. 10.3724/sp.j.1123.2013.12031 25185317

[B15] Liu Q. Gao R. Li J. Lin L. Zhao J. Sun W. (2017). Development of a genome-editing CRISPR/Cas9 system in thermophilic fungal *Myceliophthora* species and its application to hyper-cellulase production strain engineering. *Biotechnol. Biofuels* 10:1. 10.1186/s13068-016-0693-9 28053662 PMC5209885

[B16] Livak K. J. Schmittgen T. D. (2001). Analysis of relative gene expression data using real-time quantitative PCR and the 2(-Delta Delta C(T)) Method. *Methods* 25 402–408. 10.1006/meth.2001.1262 11846609

[B17] Ma M. Okuyama M. Sato M. Tagami T. Klahan P. Kumagai Y. (2017). Effects of mutation of Asn694 in *Aspergillus niger* α-glucosidase on hydrolysis and transglucosylation. *Appl. Microbiol. Biotechnol.* 101 6399–6408. 10.1007/s00253-017-8402-6 28688044

[B18] Ma M. Okuyama M. Tagami T. Kikuchi A. Klahan P. Kimura A. (2019). Novel α-1,3/α-1,4-Glucosidase from *Aspergillus niger* Exhibits Unique Transglucosylation to Generate High Levels of Nigerose and Kojibiose. *J. Agric. Food Chem.* 67 3380–3388. 10.1021/acs.jafc.8b07087 30807133

[B19] Maniglia B. C. Castanha N. Le-Bail P. Le-Bail A. Augusto P. E. D. (2021). Starch modification through environmentally friendly alternatives: A review. *Crit. Rev. Food Sci. Nutr.* 61 2482–2505. 10.1080/10408398.2020.1778633 34374585

[B20] Meng J. Chroumpi T. Makela M. R. de Vries R. P. (2022). Xylitol production from plant biomass by *Aspergillus niger* through metabolic engineering. *Bioresour. Technol.* 344(Pt A):126199. 10.1016/j.biortech.2021.126199 34710597

[B21] Nagayoshi E. Ozeki K. Mai H. Minetoki T. Takii Y. (2015). Transglycosilation activity of *Aspergillus oryzae*-derived α-glucosidase. *J. Biol. Macromol.* 15 13–27.

[B22] Nigam P. Singh D. (1995). Enzyme and microbial systems involved in starch processing. *Enzyme Microb. Technol.* 17 770–778.

[B23] Omasits U. Ahrens C. H. Muller S. Wollscheid B. (2014). Protter: Interactive protein feature visualization and integration with experimental proteomic data. *Bioinformatics* 30 884–886. 10.1093/bioinformatics/btt607 24162465

[B24] Parashar D. Satyanarayana T. (2017). Engineering a chimeric acid-stable α-amylase-glucoamylase (Amy-Glu) for one step starch saccharification. *Int. J. Biol. Macromol*. 99 274–281. 10.1016/j.ijbiomac.2017.02.083 28238910

[B25] Payne C. Methven L. Fairfield C. Bell A. (2011). Consistently inconsistent: Commercially available starch-based dysphagia products. *Dysphagia* 26 27–33. 10.1007/s00455-009-9263-7 20043180

[B26] Perez-Riverol Y. Bai J. Bandla C. García-Seisdedos D. Hewapathirana S. Kamatchinathan S. (2022). The PRIDE database resources in 2022: A hub for mass spectrometry-based proteomics evidences. *Nucleic Acids Res*. 50 D543–D552. 10.1093/nar/gkab1038 34723319 PMC8728295

[B27] Saburi W. Okuyama M. Kumagai Y. Kimura A. Mori H. (2015). Biochemical properties and substrate recognition mechanism of GH31 α-glucosidase from *Bacillus* sp. AHU 2001 with broad substrate specificity. *Biochimie* 108 140–148. 10.1016/j.biochi.2014.11.010 25450253

[B28] Song K. M. Okuyama M. Nishimura M. Tagami T. Mori H. Kimura A. (2013). Aromatic residue on ß→α loop 1 in the catalytic domain is important to the transglycosylation specificity of glycoside hydrolase family 31 α-glucosidase. *Biosci. Biotechnol. Biochem.* 77 1759–1765. 10.1271/bbb.130325 23924743

[B29] Sun Q. Xing Y. Qiu C. Xiong L. (2014). The pasting and gel textural properties of corn starch in glucose, fructose and maltose syrup. *PLoS One* 9:e95862. 10.1371/journal.pone.0095862 24755772 PMC3996010

[B30] Tomasik P. Horton D. (2012). Enzymatic conversions of starch. *Adv. Carbohydr. Chem. Biochem.* 68 59–436. 23218124 10.1016/B978-0-12-396523-3.00001-4

[B31] van Zyl W. H. Bloom M. Viktor M. J. (2012). Engineering yeasts for raw starch conversion. *Appl. Microbiol. Biotechnol.* 95 1377–1388. 10.1007/s00253-012-4248-0 22797599

[B32] Vanier N. L. El Halal S. L. M. Dias A. R. G. da Rosa Zavareze E. (2017). Molecular structure, functionality and applications of oxidized starches: A review. *Food Chem.* 221 1546–1559. 10.1016/j.foodchem.2016.10.138 27979128

[B33] Vihinen M. Mantsala P. (1989). Microbial amylolytic enzymes. *Crit. Rev. Biochem. Mol. Biol.* 24 329–418. 10.3109/10409238909082556 2548811

[B34] Wang J. Wu Y. Gong Y. Yu S. Liu G. (2015). Enhancing xylanase production in the thermophilic fungus *Myceliophthora thermophila* by homologous overexpression of *Mtxyr1*. *J. Ind. Microbiol. Biotechnol.* 42 1233–1241. 10.1007/s10295-015-1628-3 26173497

[B35] Wang L. Cao Z. Hou L. Yin L. Wang D. Gao Q. (2016). The opposite roles of *agdA* and *glaA* on citric acid production in *Aspergillus niger*. *Appl. Microbiol. Biotechnol.* 100 5791–5803. 10.1007/s00253-016-7324-z 26837219

[B36] Wong D. W. S. Robertson G. H. Lee C. C. Wagschal K. (2007). Synergistic action of recombinant α-amylase and glucoamylase on the hydrolysis of starch granules. *Protein J*. 26 159–164. 10.1007/s10930-006-9057-9 17203391

[B37] Xu G. Li J. Liu Q. Sun W. Jiang M. Tian C. (2018). Transcriptional analysis of *Myceliophthora thermophila* on soluble starch and role of regulator AmyR on polysaccharide degradation. *Bioresour. Technol.* 265 558–562. 10.1016/j.biortech.2018.05.086 29843921

[B38] Yuan X. L. van der Kaaij R. M. van den Hondel C. A. Punt P. J. van der Maarel M. J. Dijkhuizen L. (2008). *Aspergillus niger* genome-wide analysis reveals a large number of novel α-glucan acting enzymes with unexpected expression profiles. *Mol. Genet. Genomics* 279 545–561. 10.1007/s00438-008-0332-7 18320228 PMC2413074

[B39] Zhou C. Xue Y. Zhang Y. Zeng Y. Ma Y. (2009). Recombinant expression and characterization of *Thermoanaerobacter tengcongensis* thermostable α-glucosidase with regioselectivity for high yield isomaltooligosaccharides synthesis. *J. Microbiol. Biotechnol.* 19 1547–1556. 10.4014/jmb.0905.05006 20075617

