# Supplementary material for: Manipulation of an α-glucosidase in the industrial glucoamylase-producing Aspergillus niger strain O1 to decrease non-fermentable sugars production and increase glucoamylase activity
Source: Front Microbiol. 2022 Oct 20;13:1029361. doi: 10.3389/fmicb.2022.1029361 (PMC9633098; doi:10.3389/fmicb.2022.1029361)
Supplement: Supplementary file 1 [file Data_Sheet_1.docx]

***Supplementary material***

1. **Supplementary Figures and Tables**

**1.1 Supplementary Figures**

**
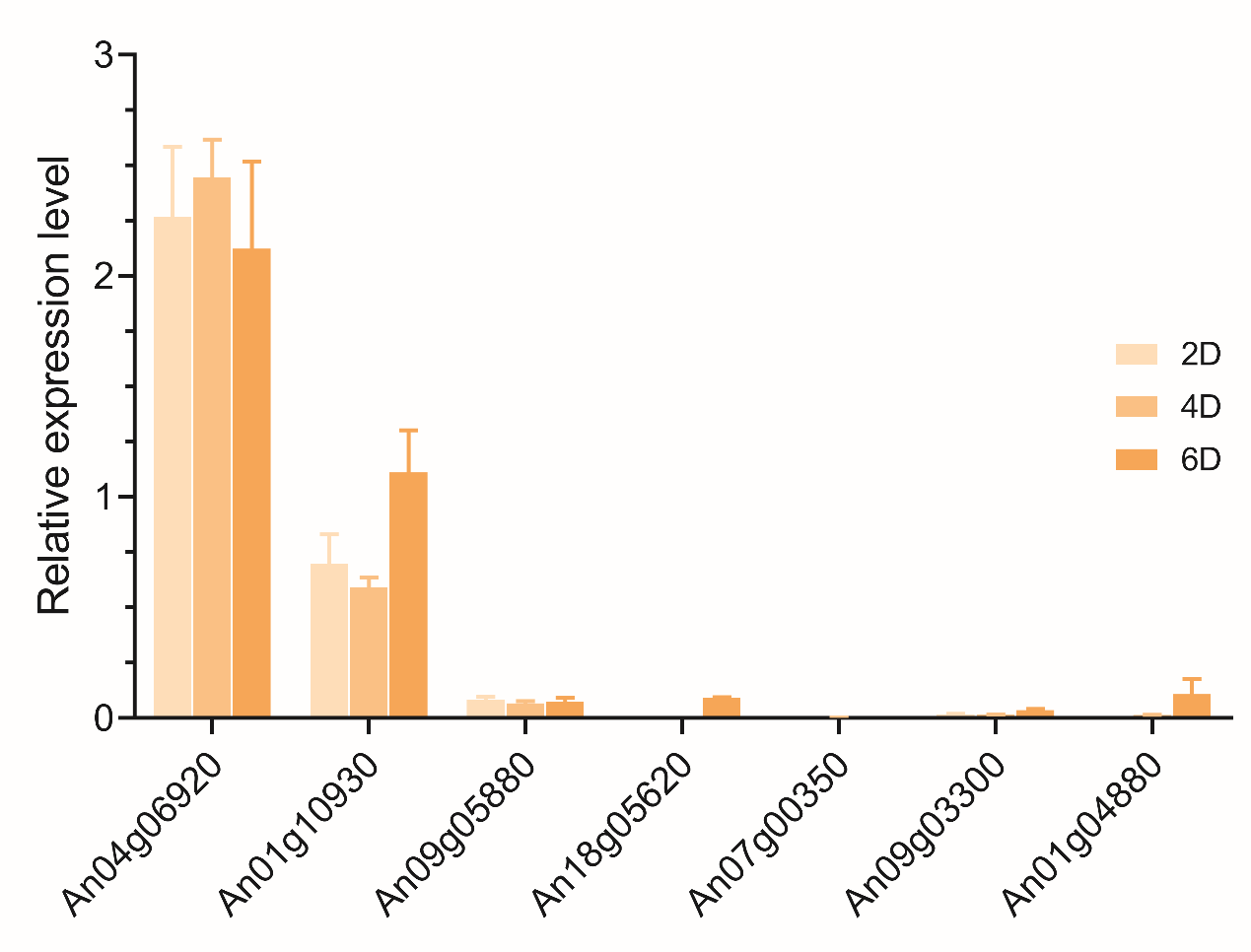
**

**Supplementary Figure 1.** The expression levels of seven potential α-glucosidase genes in 2-day, 4-day and 6-day fermentation cultures of *A. niger* O1. Values are means ± SD (*n* = 3 repeats).


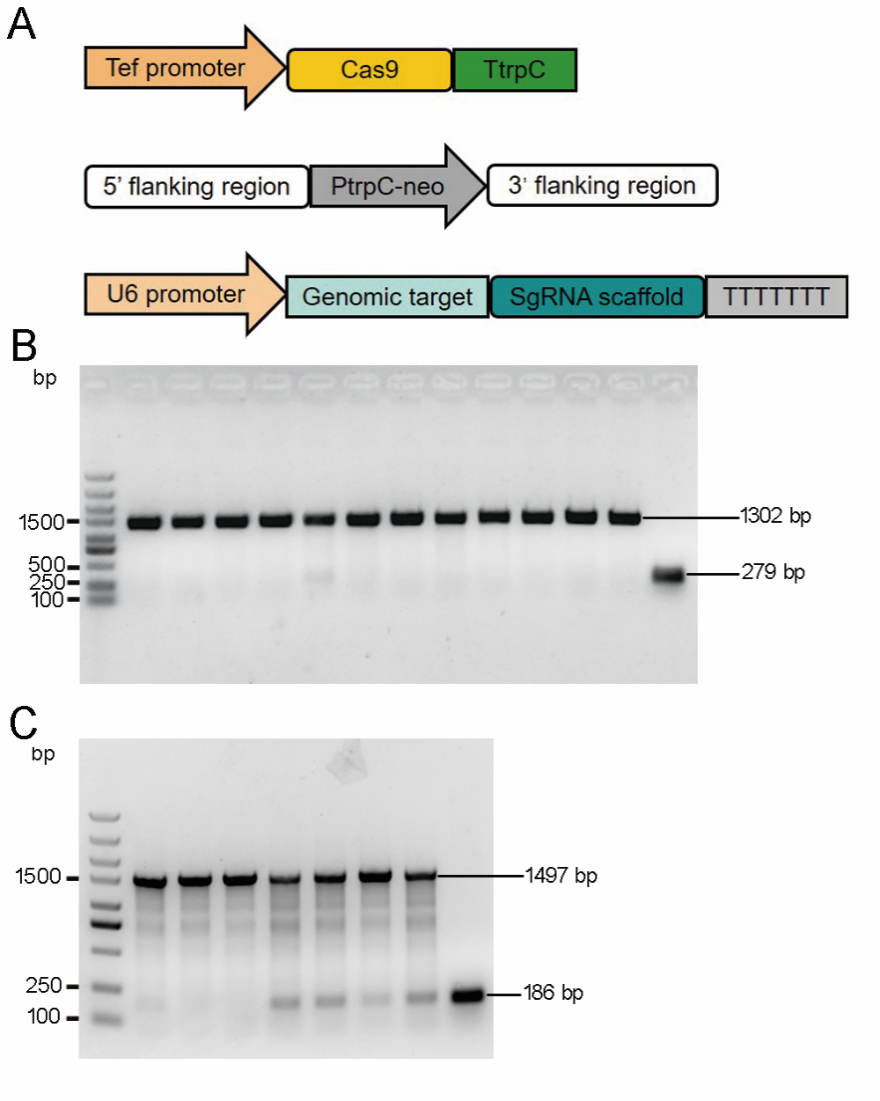


**Supplementary Figure 2.** Schematic representation of three parts of the CRISPR/Cas9 based gene-editing system for *agdA* or *agdB* knockout **(A)** and identification of *agdA* **(B)** /*agdB* **(C)** deletion transformants by diagnostic PCR. 279 bp and 186 bp represent negative, 1302 bp and 1497 bp represent the PCR products of positive *agdA* and *agdB* knockout strains, respectively.


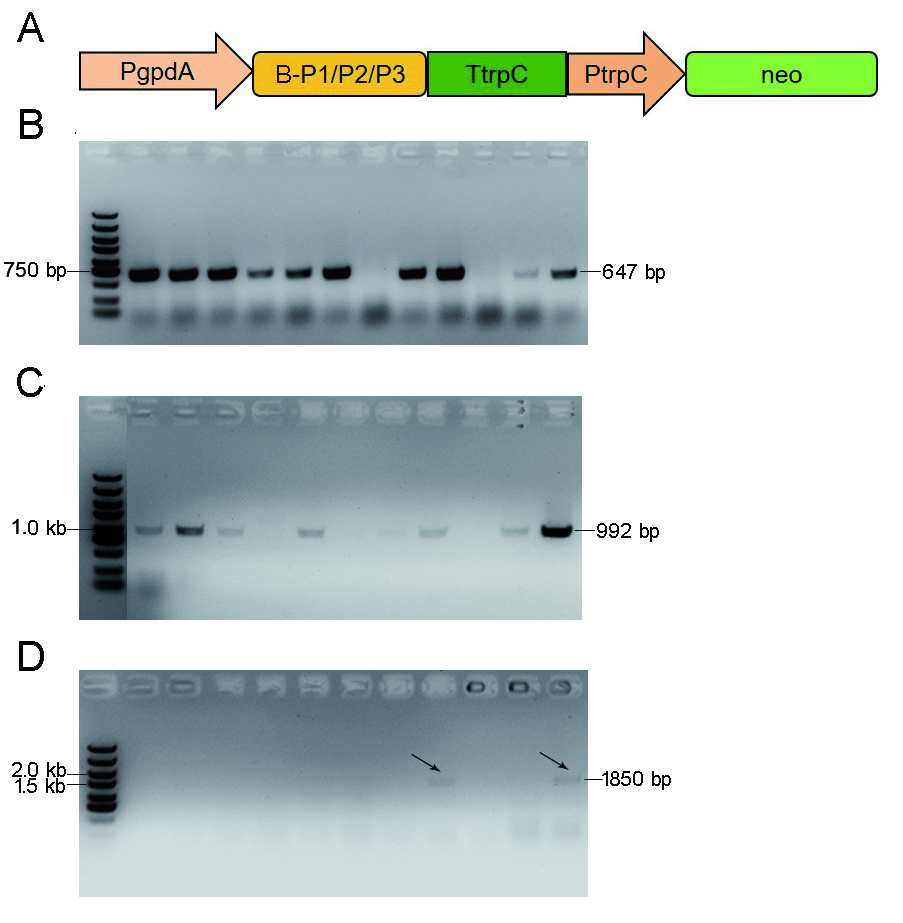


**Supplementary Figure 3.** Schematic representation of the specific domain overexpression of agdB **(A)** and identification of B-P1 **(B)**, B-P2 **(C)**, B-P3 **(D)** overexpression transformants by diagnostic PCR. 647 bp, 992 bp and 1850 bp represent the PCR products of positive strains of B-P1, B-P2 and B-P3 overexpression, respectively.


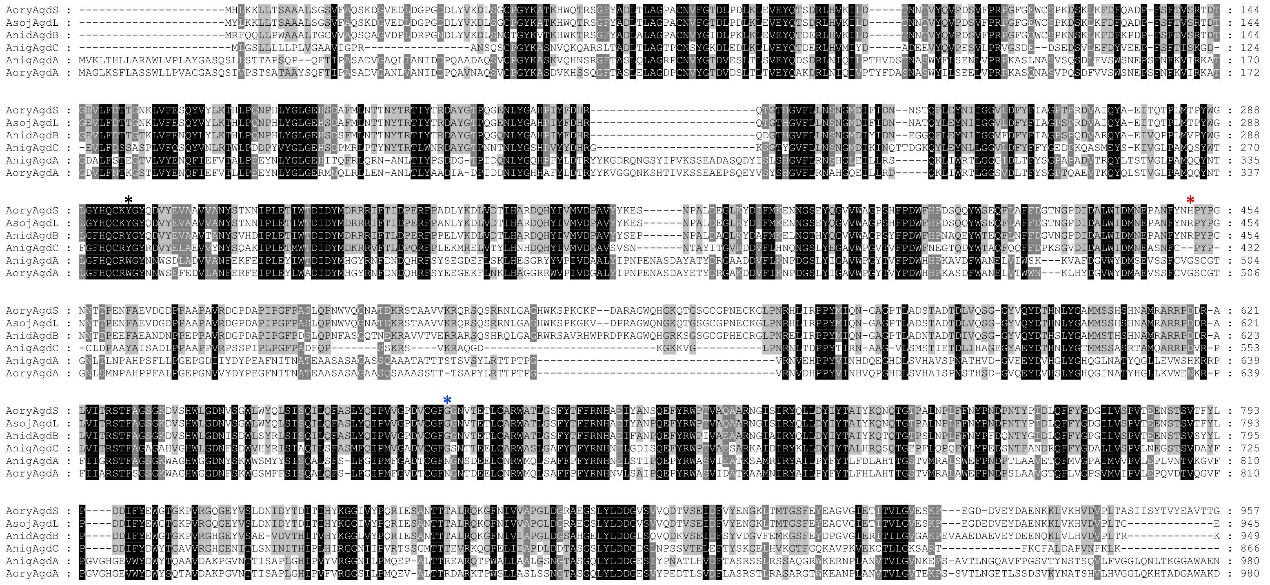


**Supplementary Figure 4.** Multiple alignment of amino acid sequences of α-glucosidases from *Aspergillus spp*. *Aory*AgdS, *Aspergillus oryzae* BAE64257; *Aory*AgdA, *Aspergillus oryzae* BAA08125; *Asoj*AgdL, A*spergillus sojae* DM849390; *Anig*, *Aspergillus niger*; *Anid*AgdB, *Aspergillus nidulans* BAB39856.Black star, Tyr296; red star, His/Arg450; blue star, Asn694.


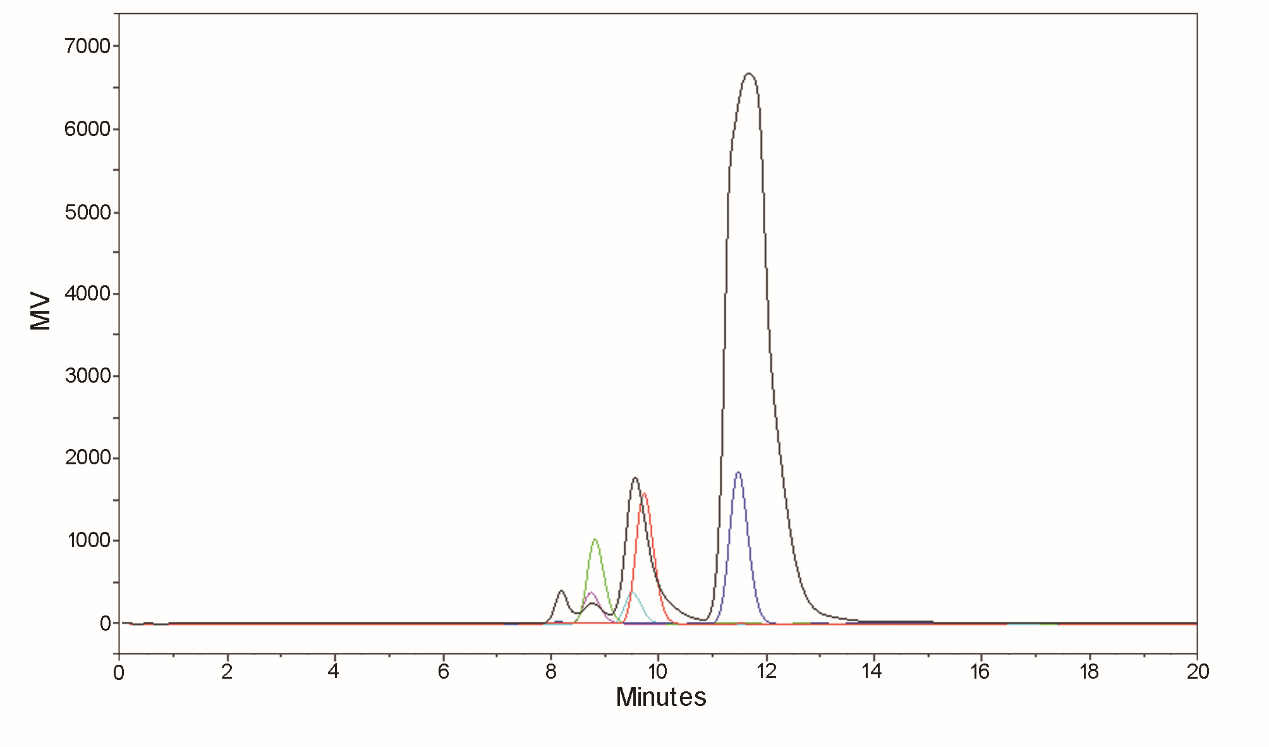


**Supplementary Figure 5.** Retention time of peaks for a variety of standards. Blue peak, glucose; red peak, maltose; cyan peak, isomaltose; green peak, maltotriose; purple peak, panose; black peaks, products of starch liquefaction and saccharification.


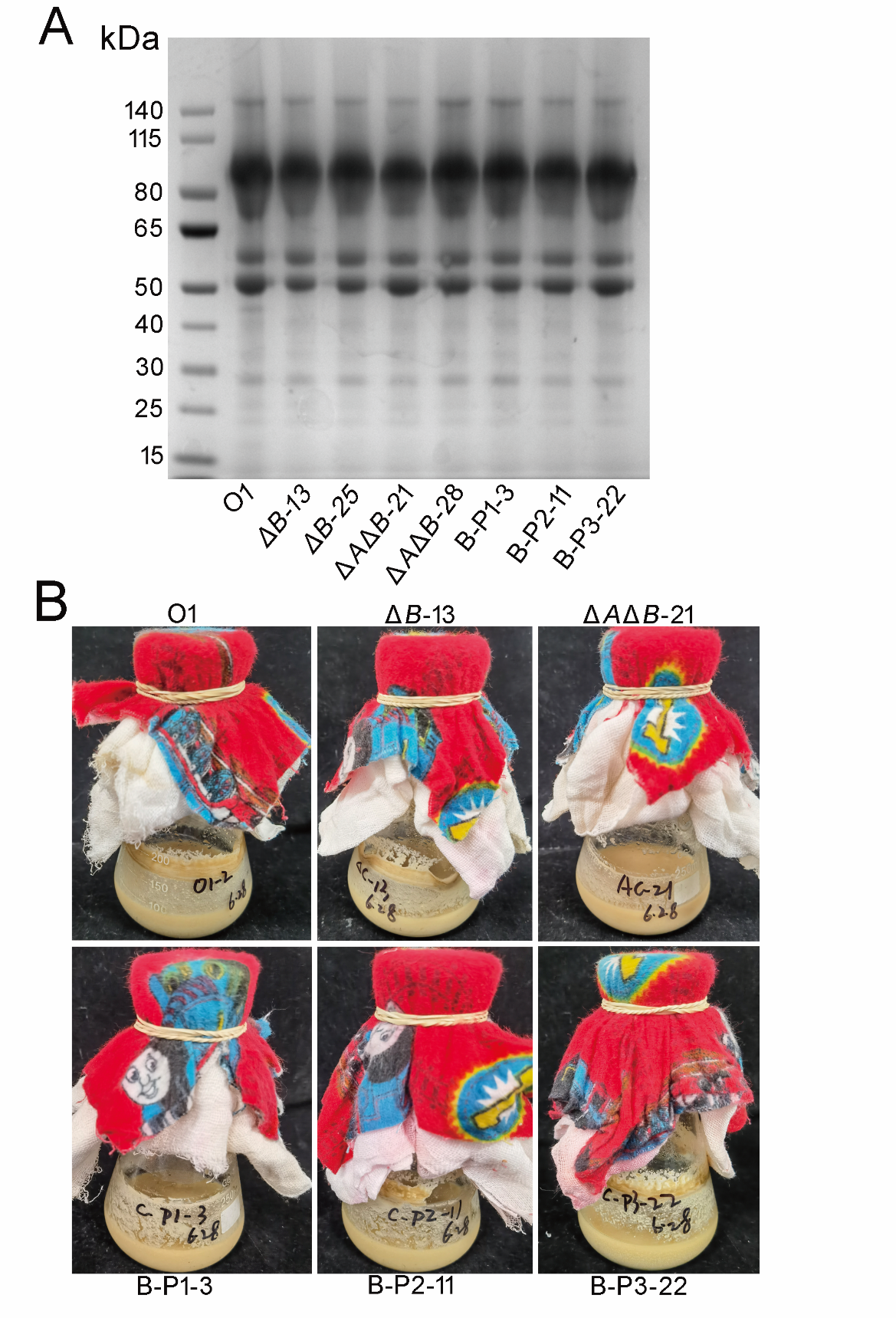


**Supplementary Figure 6.** SDS-PAGE analysis of secreted proteins in 6-day supernatants of fermentation cultures **(A)** and phenotypes of 6-day fermentation cultures **(B)** from strain O1, *agdA* or/and *agdB* knockout strains, domain-OE strains.

**1.2 Supplementary Tables**

**Supplementary Table 1.** List of PCR primers used in this study.

| primer name | sequence（5‘-3’） | function |
| --- | --- | --- |
| agdA-identy-F | CAGCCGCAGTTTACCATTCCTG | Diagnostic PCR |
| agdA-identy-R | GTTTGTGGAGTCAACATGAGTG | Diagnostic PCR |
| agdB-identy-F | CTGCTGTCATTGGACCCAG | Diagnostic PCR |
| agdB-identy-R | CAAGCCAACACTCACCAGTCT | Diagnostic PCR |
| A-donor-F1 | CATCCATGCGCTACGGAGTACTTTCGGAGAATCAG | Cloning 5’ of agdA |
| A-donor-R1 | CTTCAATATCAGTTAACGTCGGCGTCGGCAGCCTGAGGATC | Cloning 5’ of agdA |
| A-donor-F2 | GATCCTCAGGCTGCCGACGCCGACGTTAACTGATATTGAAG | Cloning of *neo* gene |
| A-donor-R2 | TATTCAGTCGATCCGAATCCTTCAGAAGAACTCGTCAAGAAG | Cloning of *neo* gene |
| A-donor-F3 | CTTCTTGACGAGTTCTTCTGAAGGATTCGGATCGACTGAATA | Cloning 3’ of agdA |
| A-donor-R3 | GACCAGATAACAAGCTCGTTAGCCCAGAAGTCAAC | Cloning 3’ of agdA |
| agdA-T1-R1 | GGACAAACCGACTGCGCGTCTTTGAATTATATAGGAATAATG | construct for sgRNA cassette |
| agdA-T1-F2 | GACGCGCAGTCGGTTTGTCCGTTTTAGAGCTAGAAATAGC | construct for sgRNA cassette |
| agdA-T2-R1 | GGTACTCCACAGACAGTGTCTTTGAATTATATAGGAATAATG | construct for sgRNA cassette |
| agdA-T2-F2 | GACACTGTCTGTGGAGTACCGTTTTAGAGCTAGAAATAGC | construct for sgRNA cassette |
| B-donor-F1 | CATCTGCATCCGACATATCGCAGGTAGAGAAGCTG | Cloning 5’ of agdB |
| B-donor-R1 | CCTCCACTAGCATTACACTTGTGACCTAGCCTGCTTTTGGAC | Cloning 5’ of agdB |
| B-donor-F2 | GTCCAAAAGCAGGCTAGGTCACAAGTGTAATGCTAGTGGAGG | Cloning of *neo* gene |
| B-donor-R2 | GCTTGAGGTCTTCCAAATCCTCTATTCCTTTGCCCTCGGACG | Cloning of *neo* gene |
| B-donor-F3 | CGTCCGAGGGCAAAGGAATAGAGGATTTGGAAGACCTCAAGC | Cloning 3’ of agdB |
| B-donor-R3 | GACACCAGGCCAAACAGCACCTGCATAGTTTAGCTC | Cloning 3’ of agdB |
| agdB-T1-R1 | CAGTCAGTGACCTAGCCTGCTTTGAATTATATAGGAATAATG | construct for sgRNA cassette |
| agdB-T1-F2 | GCAGGCTAGGTCACTGACTGGTTTTAGAGCTAGAAATAGC | construct for sgRNA cassette |
| agdB-T2-R1 | TGCCATAGCTATTACAAGGCTTTGAATTATATAGGAATAATG | construct for sgRNA cassette |
| agdB-T2-F2 | GCCTTGTAATAGCTATGGCAGTTTTAGAGCTAGAAATAGC | construct for sgRNA cassette |
| An Ptef/cas9-F | CTGGTACGGTACCAAATCTTG | Cloning of *cas9* gene |
| An Ptef/cas9-R | AAGAAGGATTACCTCTAAAC | Cloning of *cas9* gene |
| B-3amp-F | CACATCTAAACAACTAGTGATATGTTGGGGTCTTTGCTTTTAC | Cloning of B-P1/B-P2/B-P3 |
| B-P1-R | TAAGTGGATCCGAATTCGATTTAATCGGGCAACCAGGTGCGAAG | Cloning of B-P1 |
| B-P2-R | TAAGTGGATCCGAATTCGATTTACATTGCCGGGAGACCGACAATC | Cloning of B-P2 |
| B-P3-link-F | GTCCAAAAGCAGGCTAGGTCAGTCGGTCTCCCGGCAATGCAG | Cloning of B-P3 |
| B-P3-link-R | CTGCATTGCCGGGAGACCGACTGACCTAGCCTGCTTTTGGAC | Cloning of B-P3 |
| agdB-P3-R | TAAGTGGATCCGAATTCGATTCATGTCCTGACAGGTATGATAT | Cloning of B-P3 |
| agdB-OE-identy-F | CATCTTTCCCATCATCATCTC | Diagnostic PCR |
| agdB-OE-identy-R | CTGACATCGACACCAACGATC | Diagnostic PCR |
| agdA-RT-F | TGCTTCCGCAGATGTCGGTGC | Expression level detection of *agdA* |
| AgdA-RT-R | TTGTGGAGTCAACATGAGTGG | Expression level detection of *agdA* |
| agdB-RT-F | GTTGCCCAGGGTATAAGGCGTC | Expression level detection of *agdB* |
| agdB-RT-F | CACTGTCCTCAGAGTCCTCGTC | Expression level detection of *agdB* |

| 5880-RT-F | GCTGTCCTTGGTGATCCTACTCG | Expression level detection of An09g05880 |
| --- | --- | --- |
| 5880-RT-R | GAGGAAGGAGACAACGAGAGG | Expression level detection of An09g05880 |
| 5620-RT-F | GTCCCGAAGAGCGATCCTTC | Expression level detection of An18g05620 |
| 5620-RT-R | CGACATAGGAGCGAAGAGGCAG | Expression level detection of An18g05620 |
| 0350-RT-F | CTGTCCTACCAAGCAAGCCAAC | Expression level detection of An07g00350 |
| 0350-RT-R | CACCGCAATCCAGAAGATAACC | Expression level detection of An07g00350 |
| 3300-RT-F | GGTCTTCGTATCCAGCATGGT | Expression level detection of An09g03300 |
| 3300-RT-R | CTCACTTCCATCGTCATTGACG | Expression level detection of An09g03300 |
| 4880-RT-F | CTTCCATGCCCACCGAAGACTG | Expression level detection of An01g04880 |
| 4880-RT-R | CTCAAATCGCATGGTTAGGTGG | Expression level detection of An01g04880 |
| Actin-RT-F | ATTGGTATGGGTCAGAAG | Expression level detection of *actin* |
| Actin-RT-R | GAGTTCATTGTAGAAGGTG | Expression level detection of *actin* |

**2 Supplementary data**

>agdA 5’ flanking sequence of donor DNA

CATCCATGCGCTACGGAGTACTTTCGGAGAATCAGCACCCCTGCACAAAGCATTGTCAATGTGTTTTCTTATGTCAAAAGCTGACAGAGTCTGAGGCTCGCTGACGATGGGATTCATGCTAATGACGGTCCGAAAGAGCTTTCACGTAACACTGGTGAACATCCCACTCGGGAAGCCGAGACTTGTGACCTACTTAGTCAAATGAGATGATTATCAAAGCCATTAAATGCCTCGCTGTCAGGGGCCCTGGTAAGTGTCTTCATTAATCGAAACCCATCTTCATTCGTCCCCGCCTTCAGTGCTCATCATTTTAGGTTTAGAAGCAAGATTGAGTGCCACCTGCTTTACAAACCAGCATGGGTAGTCTGCTGTTGAAATTCTTCACCGGGAGCATTCTGGGGAAGGTGCAAAAGGCGGCGCGAAGTGGTCGGGTCGCGATTGTAGTCTGGATTGGAGCACAAGAATCGTCAGAGCCGAAGCCCGAACTGAGGGGGTCTCGGTCATTTATCGGGATGAGAGCCAATCAGCGTGCGCTCATCATCTGATCGTCTGGCTGCCAGGCCCCTCAGGCATCAATACGGTACTCGGCAGTATCCACTCCCGTTTCTCCGGTGCAACAAATCATCGTTGGAGAATCCCCAGCTCCCCCGCCAACTGGGGTCGATGCTTCTCCAGTTGTCCTGGTTTCTCCCATGAACTCGCTTACGATAAGCTGCTGTACCAGCCCACCAGCACAACAATATCTTCAATCAGGTAGGTGCTTGTTCGTTACCTGCCCCATCCTCTCCTCTTCTTCGGTCATTATGAACTCAATTCGGTCGCTAGCTTTGCCGATTCTCCGCAGTCCATAAAAATATATCTGCATTTGCCCCTTACACGTCGGGAATTCACCGGCGCAATGAGCCTTCGGGTATGGTCGCACAGCGTCATGTCAATAGGAGGCTGCTCCTAGTGGTGATCTACTAGTCGCCTCAACACAGCAATATATAAATAACAAGAGCATTCCTTGAGCACATCTGGGTAATAGCTGTTCCATTCTCATCAAGGATTACGCGACCGTGCCTCGAGCCTCCTTAAGCGAGCCATGGTGAAGTTGACGCATCTCCTTGCCAGAGCATGGCTTGTCCCTCTGGCTTATGGAGCGAGCCAGTCACTCTTATCCACCACTGCCCCTTCGCAGCCGCAGTTTACCATTCCTGCTTCCGCAGATGTCGGTGCGCAGCTGATTGCCAACATCGATGATCCTCAGGCTGCCGACGC

>agdA 3’ flanking sequence of donor DNA

AGGATTCGGATCGACTGAATATTCAGATTCTCCCCACTCATGTTGACTCCACAAACGCTTCTTGGTACTTTCTTTCGGAAAACCTGGTCCCCAGACCCAAGGCTTCCCTCAATGCATCTGTATCCCAGAGCGACCTTTTTGTGTCATGGTCAAATGAGCCGTCGTTCAATTTCAAGGTGATCCGAAAGGCTACAGGCGACGCGCTTTTCAGTACAGAAGGCACTGTGCTCGTATATGAGAATCAGTTCATCGAATTTGTGACCGCGCTCCCTGAAGAATATAACTTGTATGGCCTTGGGGAGCATATCACGCAATTCCGCCTCCAGAGAAATGCTAATCTGACCATATATCCTTCGGATGATGGAACACCTATTGACCAGTGAGTACTGATATCCCGCCCGTATCTTCTGGTTCTACTCTTGAAACTTACTCGTCCTAGAAACCTCTACGGCCAACATCCCTTCTATCTGGATACAAGATATTACAAAGGAGATAGGCAGAATGGGTCTTATATTCCCGTCAAAAGCAGCGAGGCTGATGCCTCGCAAGATTATATCTCCCTCTCTCATGGCGTGTTTCTGAGGAACTCTCATGGACTTGAGATACTCCTCCGGTCTCAAAAATTGATCTGGCGGACCCTAGGTGGAGGAATCGATCTCACCTTCTACTCAGGCCCCGCCCCGGCCGATGTTACCAGGCAATATCTTACCAGCACTGTGGGATTACCGGCCATGCAGCAATACAACACTCTTGGATTCCACCAATGTCGTTGGGGCTACAACAACTGGTCGGATCTGGCGGACGTTGTTGCGAACTTTGAGAAGTTTGAGATCCCGTTGGAATATATCTGGTGCGTATTGTACTGGTTTATGGTATCTCAAAACAGTCTAACAGGCACTTAGGACCGATATTGACTACATGCACGGATATCGCAACTTTGACAACGATCAACATCGCTTTTCCTACAGTGAGGGCGATGAATTTCTCAGCAAGCTACATGAGAGTGGACGCTACTATGTACCCATTGTTGATGCGGCGCTCTACATTCCTAATCCCGAAAATGCCTCTGATGCGTAAGTGTCTAGTGACAAATTATATTACTGCCTGTATGCTAATTAGCGATACAGATACGCTACGTATGACAGAGGAGCTGCGGACGACGTCTTCCTCAAGAATCCCGATGGTAGCCTCTATATTGGAGCCGTTTGGCCAGGATATACAGTCTTCCCCGATTGGCATCATCCCAAGGCAGTTGACTTCTGGGCTAACGAGCTTGTTATCTGGTC

> *agdB* 5’ flanking sequence of donor DNA

CATCTGCATCCGACATATCGCAGGTAGAGAAGCTGTCTCTCATCGCATTGACAAGCCTTTGCATAGAATGAAGCGGCAGAGTAACCCAGAGCTGCAGCTTGACTGAACACTTTGTCTTAACGCATCTGCACACTTCCCCCAGGTCCCCAAGCTTCTGCCAGGGTACCCGGCCTGCTGGAGGAGTGAATATGCACACCTCGAGTGCCACATTAATTAGTACGATGTGGTCAATACCTTATGCCACCTCATTGCTTAATGCTTGCAGCACTGGCAGTAAGCGACCGCCTCTTGACAGGTCAGAGCCACCTATCAGCTACAACTACGTATCAGCAGACCGGATCCGTTGCATCCGACGCTTTGTCTGACTCTTGTGCGGTTTTTGAGTGACCAGAATATTTACTTTGGTCCATGTTCTTTGAGTGAGATCTGATCCTGATCCTTTGAATGTCTCAGTTTGTTTGTTTGCGGGGCGTCAGGCGGGGCACGTGGTGGGGAGAGTGAGGAGAGCCACCATCACTGTCACTTCCTCGATTCCATCTCCATACTACTATTCGCTACCAAAAGCTTACTTGCATAAAATTTGGAGCCAATCATCCAGGGCATTACCGAGAGATATACCGATTAGACGTGCCCAGAGTCTAGGCTGCTGCACGAGATTCAATGAACGAGAATCGGTGTAAGTGACCTGATGTTATTTGGTCCCTGCATCAGCCCCAAGCCGATAGCGGCGAAGATCCCCGATAATTGACCGAGATGGGACGACCTTAGACTCACATTGTCTTCTTTAGGCAATCGTCTCCACGTTTCTCGGCTTTTCTATCCTATAAATATTGCTTTTTGTTTTTCCTCATAGAACTGCTCGGCTCATCCCGCCTCTTTGTCAGATACATTCCTTGGCTTCGCTGATTGAATCTGCGGGGTCCGGTCATACCGCGCAACGCCACATTATGCACTTCGGCCAACGCGCCATGCATTCAATGTCATCAGTCCGTGCCCAAACACATATAAGCCGCTGGGACCACCCAGCTGGGATATGAAGTCACGGCTTGCTGTAATCCGGGGTGATCCCAGAGCCAACATCATAATGTTGGGGTCTTTGCTTTTACTCTTACCCCTTGTGGGCGCTGCTGTCATTGGACCCAGGGCAAACAGTCAGAGTTGCCCAGGGTATAAGGCGTCCAACGTCCAAAAGCAGGCTAGGTCAC

>*agdB* 3’ flanking sequence of donor DNA

AGGATTTGGAAGACCTCAAGCTGCTTGTGGAATATCAGACTGGTGAGTGTTGGCTTGTGTGAATCAAGAGTTCCTGACTAAATGCTTGCTCAGATGAACGGTTACATGTTATGATCTACGATGCCGACGAGGAAGTCTATCAAGTTCCTGAATCAGTCCTTCCTCGCGTGGGTAGTGACGAGGACTCTGAGGACAGTGTTTTGGAATTTGACTATGTGGAAGAACCGTTTTCATTCACCATCTCCAAGGGAGATGAGGTCCTGTTTGACTCTTCGGCATCACCACTAGTTTTTCAGTCGCAATATGTGAACCTTCGCACCTGGTTGCCCGATGATCCCTATGTGTATGGTCTCGGAGAGCATTCTGACCCTATGCGCTTGCCAACATACAATTACACGCGGACCCTTTGGAACCGCGACGCGTATGGCACTCCAAACAACACCAACTTGTACGGTAGTCATCCTGTCTACTATGATCACCGTGGAAAGTCCGGAACTTATGGAGTCTTCCTGCTGAACTCTAATGGTATGGACATCAAGATCAACCAAACGACAGATGGAAAGCAGTACTTGGAATACAATCTTCTCGGCGGTGTTCTGGACTTCTACTTCTTCTACGGAGAAGATCCTAAGCAAGCGAGCATGGAATACTCAAAGATTGTCGGTCTCCCGGCAATGCAGAGTTACTGGACTTTCGGCGTATGCCCCCCACCCCCTAATCCCATAACAGTCCGAGTTGTATGCTGACTCTTCAGTTCCATCAATGCCGTTATGGATACCGCGATGTGTATGAACTTGCCGAGGTGGTCTACAACTACAGCCAGGCAAAGATTCCTCTGGAGACGATGTGGACAGATATCGACTACATGGACAAGAGAAGGGTGTTTACCCTTGATCCTCAGAGGTTCCCGCTCGAAAAGATGCGGGAGTTGGTAACCTACCTGCACAATCATGATCAGCATTACATTGTCATGGTTGACCCGGCTGTGAGCGTAAGCAGTGAGTGACTTGACGATTCCCCATCCTTGCAACTTTCAGCTAATGGATACTTTCTAGATAACACGGCATATATCACCGGCGTGAGAGACGATGTTTTCCTTCACAATCAGAACGGTAGCCTATACGAGGGTAAGTATATACACATCTCATATCTCTCAACACGAGCTAAACTATGCAGGTGCTGTTTGGCCTGGTGTC

>Whole sequence of agdB-P1

ATGTTGGGGTCTTTGCTTTTACTCTTACCCCTTGTGGGCGCTGCTGTCATTGGACCCAGGGCAAACAGTCAGAGTTGCCCAGGGTATAAGGCGTCCAACGTCCAAAAGCAGGCTAGGTCACTGACTGCGGATCTGACTCTAGCTGGTACGCCTTGTAATAGCTATGGCAAGGATTTGGAAGACCTCAAGCTGCTTGTGGAATATCAGACTGATGAACGGTTACATGTTATGATCTACGATGCCGACGAGGAAGTCTATCAAGTTCCTGAATCAGTCCTTCCTCGCGTGGGTAGTGACGAGGACTCTGAGGACAGTGTTTTGGAATTTGACTATGTGGAAGAACCGTTTTCATTCACCATCTCCAAGGGAGATGAGGTCCTGTTTGACTCTTCGGCATCACCACTAGTTTTTCAGTCGCAATATGTGAACCTTCGCACCTGGTTGCCCGATTGA

> Whole sequence of agdB-P2

ATGTTGGGGTCTTTGCTTTTACTCTTACCCCTTGTGGGCGCTGCTGTCATTGGACCCAGGGCAAACAGTCAGAGTTGCCCAGGGTATAAGGCGTCCAACGTCCAAAAGCAGGCTAGGTCACTGACTGCGGATCTGACTCTAGCTGGTACGCCTTGTAATAGCTATGGCAAGGATTTGGAAGACCTCAAGCTGCTTGTGGAATATCAGACTGATGAACGGTTACATGTTATGATCTACGATGCCGACGAGGAAGTCTATCAAGTTCCTGAATCAGTCCTTCCTCGCGTGGGTAGTGACGAGGACTCTGAGGACAGTGTTTTGGAATTTGACTATGTGGAAGAACCGTTTTCATTCACCATCTCCAAGGGAGATGAGGTCCTGTTTGACTCTTCGGCATCACCACTAGTTTTTCAGTCGCAATATGTGAACCTTCGCACCTGGTTGCCCGATGATCCCTATGTGTATGGTCTCGGAGAGCATTCTGACCCTATGCGCTTGCCAACATACAATTACACGCGGACCCTTTGGAACCGCGACGCGTATGGCACTCCAAACAACACCAACTTGTACGGTAGTCATCCTGTCTACTATGATCACCGTGGAAAGTCCGGAACTTATGGAGTCTTCCTGCTGAACTCTAATGGTATGGACATCAAGATCAACCAAACGACAGATGGAAAGCAGTACTTGGAATACAATCTTCTCGGCGGTGTTCTGGACTTCTACTTCTTCTACGGAGAAGATCCTAAGCAAGCGAGCATGGAATACTCAAAGATTGTCGGTCTCCCGGCAATGTGA

> Whole sequence of agdB-P3

ATGTTGGGGTCTTTGCTTTTACTCTTACCCCTTGTGGGCGCTGCTGTCATTGGACCCAGGGCAAACAGTCAGAGTTGCCCAGGGTATAAGGCGTCCAACGTCCAAAAGCAGGCTAGGTCAGTCGGTCTCCCGGCAATGCAGAGTTACTGGACTTTCGGCTTCCATCAATGCCGTTATGGATACCGCGATGTGTATGAACTTGCCGAGGTGGTCTACAACTACAGCCAGGCAAAGATTCCTCTGGAGACGATGTGGACAGATATCGACTACATGGACAAGAGAAGGGTGTTTACCCTTGATCCTCAGAGGTTCCCGCTCGAAAAGATGCGGGAGTTGGTAACCTACCTGCACAATCATGATCAGCATTACATTGTCATGGTTGACCCGGCTGTGAGCGTAAGCAATAACACGGCATATATCACCGGCGTGAGAGACGATGTTTTCCTTCACAATCAGAACGGTAGCCTATACGAGGGTGCTGTTTGGCCTGGTGTCACTGTTTTCCCAGACTGGTTCAATGAGGGTACTCAGGATTACTGGACTGCGCAATTTCAACAGTTCTTTGATCCCAAGTCCGGAGTCGATATTGACGCCCTGTGGATTGACATGAACGAAGCCTCCAATTTCTGCCCTTATCCTTGTCTGGACCCAGCGGCATACGCGATCTCCGCCGACCTCCCACCGGCAGCACCACCTGTTCGGCCAAGCAGCCCGATCCCACTGCCCGGATTCCCCGCGGACTTTCAGCCTTCGTCTAAGCGATCTGTTAAAAGAGCGCAAGGAGATAAAGGGAAGAAGGTTGGGTTGCCCAATCGCAACCTCACTGACCCGCCCTACACCATTCGGAATGCCGCAGGTGTCCTTAGTATGAGCACTATCGAGACGGATCTCATTCATGCGGGTGAAGGGTATGCCGAGTATGATACTCACAATCTCTATGGAACAATGATGAGCTCTGCTTCCCGCACGGCTATGCAGGCCCGCCGTCCCGATGTGAGGCCTTTGGTCATCACTCGCAGTACGTTTGCAGGCGCTGGAGCACACGTAGGACACTGGCTGGGCGACAACTTTAGCGATTGGGTTCACTACCGGATCTCCATCGCGCAGATCCTCTCCTTCGCGTCCATGTTCCAGATTCCAATGGTCGGGGCTGACGTGTGTGGGTTTGGTAGCAACACGACGGAGGAATTGTGTGCCCGATGGGCGTCACTTGGTGCCTTCTATACGTTCTACCGCAATCATAACGAGCTGGGCGACATATCGCAAGAGTTCTACCGCTGGCCTACGGTTGCCGAGTCCGCGCGTAAGGCCATTGACATCCGGTACAAGCTCCTCGATTATATCTACACTGCTCTTCACCGGCAAAGCCAGACCGGCGAGCCATTCCTGCAGCCTCAATTCTACCTGTACCCTGAGGATTCGAACACCTTTGCGAACGACCGGCAGTTCTTCTATGGTGACGCCCTTCTTGTCAGCCCCGTGTTGAATGAGGGATCCACCTCAGTCGACGCATACTTCCCGGACGACATCTTCTACGATTGGTACACAGGGGCAGTGGTGCGTGGGCACGGAGAAAACATCACGCTCAGCAACATCAACATCACCCACATCCCTCTGCACATCCGCGGTGGAAATATCATACCTGTCAGGACATGA

>agdA-sgRNA-cassette-1

Cagttacttataagcttggagcttggatctctttgaggtggaccttccttgaagggtttcatctctgtactatcatgcgaatgctaaagcagaactttaacagaaccaccagtgtctaataaattcgatccgtatattgtgcaccattactcatctgtgtttcccccaaacatgcagtctcctgcgcagatagactgtcaactatagtaattcccgtccgcgaagccgccctatccaaaagtgtattaccctctcttgtatgcaacaagagtcgttctttctcgcgctaatacccatccgtctatcgcacaattaaaccttctgatccctacaatttgcctgacaaaataaatgaagttcaacgtgcaaacaagctagagccagtgtacattgagtatcatctgcagctctactcaaggtactatagtacctcagccaatttgatgttcctgccttcccgcccctcgcttagccgaccaattagagttcgttaattctaaccattattcctatataattcaaagacgcgcagtcggtttgtccgttttagagctagaaatagcaagttaaaataaggctagtccgttatcaacttgaaaaagtggcaccgagtcggtgctttttt

>agdA-sgRNA-cassette-2

Cagttacttataagcttggagcttggatctctttgaggtggaccttccttgaagggtttcatctctgtactatcatgcgaatgctaaagcagaactttaacagaaccaccagtgtctaataaattcgatccgtatattgtgcaccattactcatctgtgtttcccccaaacatgcagtctcctgcgcagatagactgtcaactatagtaattcccgtccgcgaagccgccctatccaaaagtgtattaccctctcttgtatgcaacaagagtcgttctttctcgcgctaatacccatccgtctatcgcacaattaaaccttctgatccctacaatttgcctgacaaaataaatgaagttcaacgtgcaaacaagctagagccagtgtacattgagtatcatctgcagctctactcaaggtactatagtacctcagccaatttgatgttcctgccttcccgcccctcgcttagccgaccaattagagttcgttaattctaaccattattcctatataattcaaagacactgtctgtggagtaccgttttagagctagaaatagcaagttaaaataaggctagtccgttatcaacttgaaaaagtggcaccgagtcggtgctttttt

>agdB-sgRNA-cassette-1

Cagttacttataagcttggagcttggatctctttgaggtggaccttccttgaagggtttcatctctgtactatcatgcgaatgctaaagcagaactttaacagaaccaccagtgtctaataaattcgatccgtatattgtgcaccattactcatctgtgtttcccccaaacatgcagtctcctgcgcagatagactgtcaactatagtaattcccgtccgcgaagccgccctatccaaaagtgtattaccctctcttgtatgcaacaagagtcgttctttctcgcgctaatacccatccgtctatcgcacaattaaaccttctgatccctacaatttgcctgacaaaataaatgaagttcaacgtgcaaacaagctagagccagtgtacattgagtatcatctgcagctctactcaaggtactatagtacctcagccaatttgatgttcctgccttcccgcccctcgcttagccgaccaattagagttcgttaattctaaccattattcctatataattcaaagcaggctaggtcactgactggttttagagctagaaatagcaagttaaaataaggctagtccgttatcaacttgaaaaagtggcaccgagtcggtgctttttt

>agdB-sgRNA-cassette-2

Cagttacttataagcttggagcttggatctctttgaggtggaccttccttgaagggtttcatctctgtactatcatgcgaatgctaaagcagaactttaacagaaccaccagtgtctaataaattcgatccgtatattgtgcaccattactcatctgtgtttcccccaaacatgcagtctcctgcgcagatagactgtcaactatagtaattcccgtccgcgaagccgccctatccaaaagtgtattaccctctcttgtatgcaacaagagtcgttctttctcgcgctaatacccatccgtctatcgcacaattaaaccttctgatccctacaatttgcctgacaaaataaatgaagttcaacgtgcaaacaagctagagccagtgtacattgagtatcatctgcagctctactcaaggtactatagtacctcagccaatttgatgttcctgccttcccgcccctcgcttagccgaccaattagagttcgttaattctaaccattattcctatataattcaaagccttgtaatagctatggcagttttagagctagaaatagcaagttaaaataaggctagtccgttatcaacttgaaaaagtggcaccgagtcggtgctttttt

Blue letters indicate the U6 promoter. Orange letters indicate the sgRNA scaffold. Red letters indicate the target sequence of *agdA* and *agdB* genes, respectively.
